# Draft genome sequence data on *Bacillus safensis* U41 isolated from soils of Santiniketan, India

**DOI:** 10.1016/j.dib.2024.110547

**Published:** 2024-05-21

**Authors:** Binoy Kumar Show, Andrew B. Ross, Raju Biswas, Shibani Chaudhury, Srinivasan Balachandran

**Affiliations:** a Department of Environmental Studies, Siksha-Bhavana, Visva-Bharati, Santiniketan 731235, West Bengal, India; b School of Chemical and Process Engineering, University of Leeds, Leeds, LS2 9JT, United Kingdom; c Department of Botany, Siksha-Bhavana, Visva-Bharati, Santiniketan 731235, West Bengal, India

**Keywords:** Cellulase, Enzyme, Lignocellulose, *Bacillus safensis* U41

## Abstract

The draft genome sequence of an isolate of *Bacillus safensis* U41 from the soils of Santiniketan (23040′12″ N and 87039′52″ E) is reported here. *Bacillus safensis* is a bacterium that produces cellulases, which is essential for the breakdown of plant biomass. As such, it is a valuable source of digestive enzymes from plant biomass, especially cellulases. The genomic DNA was extracted from a single colony using a QIAgen Blood and Tissue kit (QIAgen Inc., Canada). Sequencing was performed via Illumina HiSeq X using 2 × 150 paired-end chemistry, generating 7,352,576 reads with sequence coverage of 509x. The assembly produced 20 contigs over 200 base pairs (bp) in length, with an N50 value of 901304 and an L50 of 2. The genome size was 3,732,407 bp, and the average GC content was 41.43 %. Genome annotation and gene predictions were performed using Prokka v.1.14.6, which identified 3783 coding sequences, 64 tRNA genes, and 3 rRNA genes.

Specifications Table

**Table:** 

Subject	Microbiology • Applied Microbiology
Specific subject area	Omics: Genomics
Type of data	Table, Figure Raw, Analyzed, Filtered, Deposited
Data collection	The genomic DNA was extracted from a single colony using a QIAgen Blood and Tissue kit (QIAgen Inc., Canada). Sequencing was performed by Illumina HiSeq X. After FastQC check, trimming and size selection were performed by Cutadapt v2.9, the de novo assembly was performed by Unicycler v0.4.4, QUAST v.5.1.0 for assembly summary statistics, assembly pipeline using BayesHammer and assembly performed with SPAdes v3.13.0, Prokka v.1.14.6 used for genome annotation and gene predictions, and the PANZER webserver used for functional annotation of the proteins.
Data source location	The U41 strain was isolated from soils of Santiniketan, West Bengal, India (23.67760N, 87.68530 E).
Data accessibility	Repository name: NCBI (National Center for Biotechnology Information) GenBank Nucleotide database Data identification number: BioProject accession number PRJNA890884 Direct URL to data: https://www.ncbi.nlm.nih.gov/bioproject/PRJNA890884 https://www.ncbi.nlm.nih.gov/sra/SRR22254258

## 1 Value of the Data

• The *B. safensis* U41 draft genome sequence may be useful for research on the taxonomy and ecology of bacteria, especially regarding taxonomic identification and dispersion.
• The researchers working in the fields of environmental microbiology, environmental biotechnology, genomics, and renewable energy may find value in the information provided in this article.
• This genomic sequence data of *B. safensis* strain U41 may be useful to researchers wishing to perform comparative genomic analysis between strains and environments.

## 2 Background

*Bacillus safensis* is a common Gram-positive, aerobic, spore-forming Bacillus found in soil [1]. Enzyme-based biodegradation can be a choice for developing appropriate methods for adequately utilizing biomass in a productive formulation. The plant biomasses used as substrates in bioenergy production comprise lignin, cellulose, and hemicelluloses. Cellulose shapes the principal portion of lignocellulose, surrounded by a hemicellulose matrix and the exterior by a lignin layer [2]. Cellulose consists of more than 100–140,000 d-glucose units and is condensed by β-1,4-glycosidic bonds or linkages and forms a straight-chain polymer. The occurrence of multiple hydroxyl groups on the glucose forms hydrogen bonds that hold the chain and make it steadier. Cellulose is hydrophilic, but its sizeable polymeric structure renders it less soluble in water [3,4]. In this work, the draft genome of cellulase-producing *B. safensis* U41 strain has been sequenced and analysed.

## 3 Data Description

The article presents the whole genome sequencing information of *Bacillus safensis* U41 and its cellulases gene, which is essential for the breakdown of plant biomass.

**Fig. 1:**
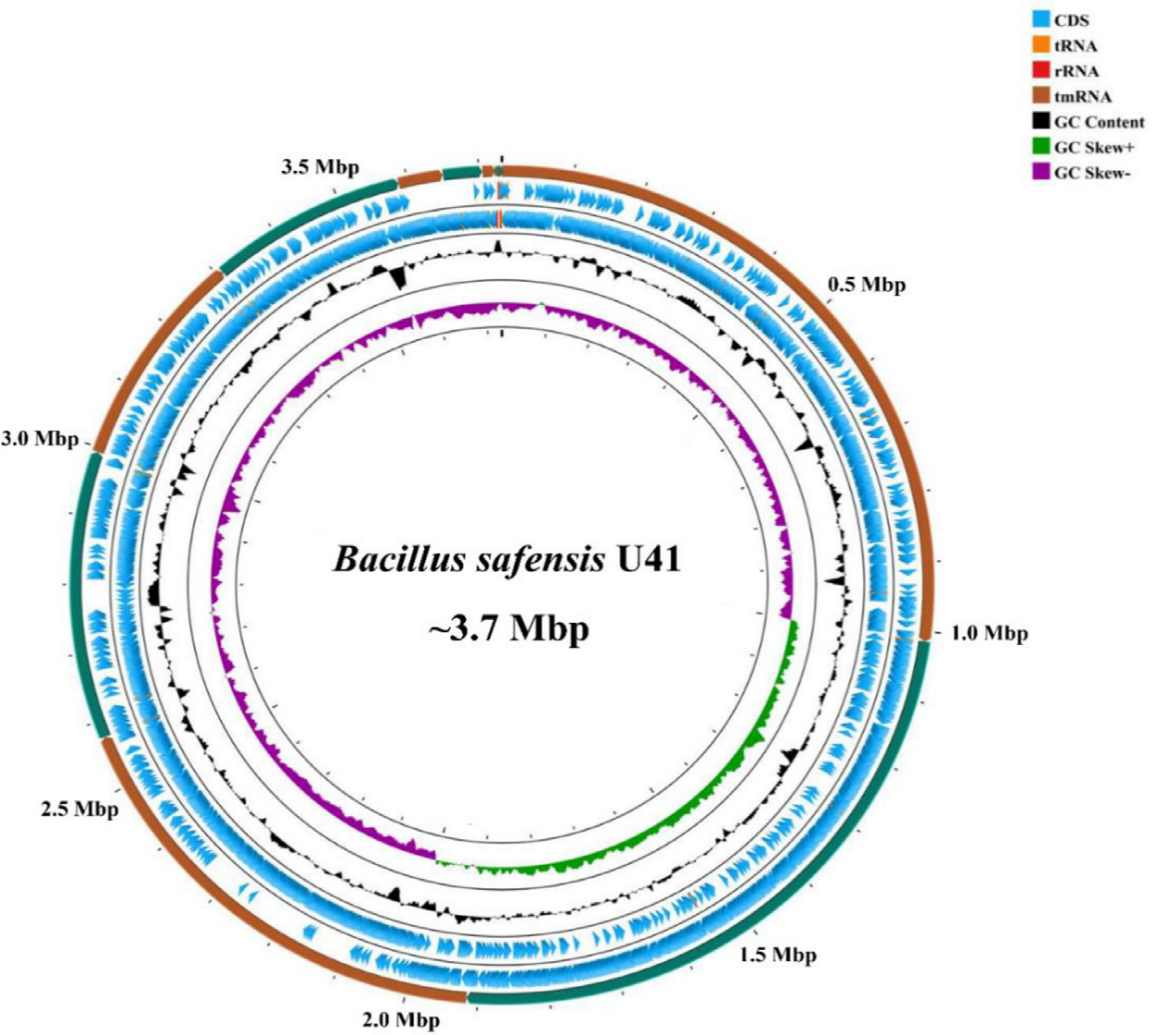
Graphical presentation of strain U41 genome (∼3.7 mb) performed with CG view server. The 6 concentric circles represent the following (from outermost to innermost): circles 2 contigs; circle 3 and 4, protein-coding genes on forward and reverse strands; circle 5 (black): G + C content; circle 6: G + C skew; circle 1: DNA base position (Mbp).

The assembly produced 20 contigs over 200 base pairs (bp) in length, with an N50 value of 901,304 and an L50 of 2. The genome shows a near-complete with low contamination (99.90% completeness and 0.20 % contamination), and sequencing coverage of 509x. The genome size was 3,732,407 bp, and the average GC content was 41.43 % (Fig. 1). Genome annotation produced 3783 CDS (coding sequences), 64 tRNA genes, and 3 rRNA genes (Table 1).

**Table 1:** General genomic features of *Bacillus safensis* U41.

Feature	Value
Genome size (bp)	3732,407
G + C content (%)	41.43
No. of contigs	20
N50	901,304
L50	2
CDS (coding sequences)	3783
tRNA	64
rRNA	3

**Fig. 2:**
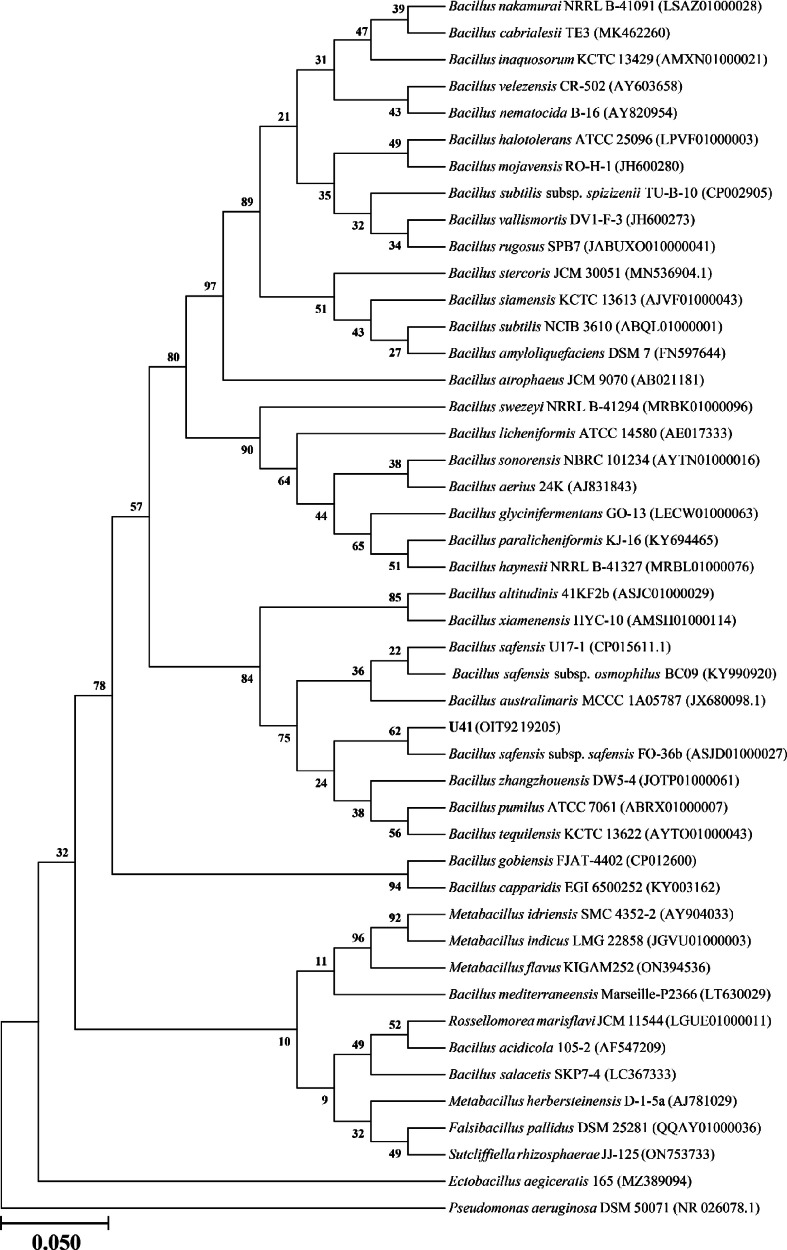
Construction of maximum likelihood phylogenetic tree elicited from the 16S rRNA gene sequences of *Bacillus safensis* U41 and the type strains of Bacillus sp. Bootstrap values (>50 % are expressed as percentages of 1000 replications) are shown at branching points.

**Fig. 3:**
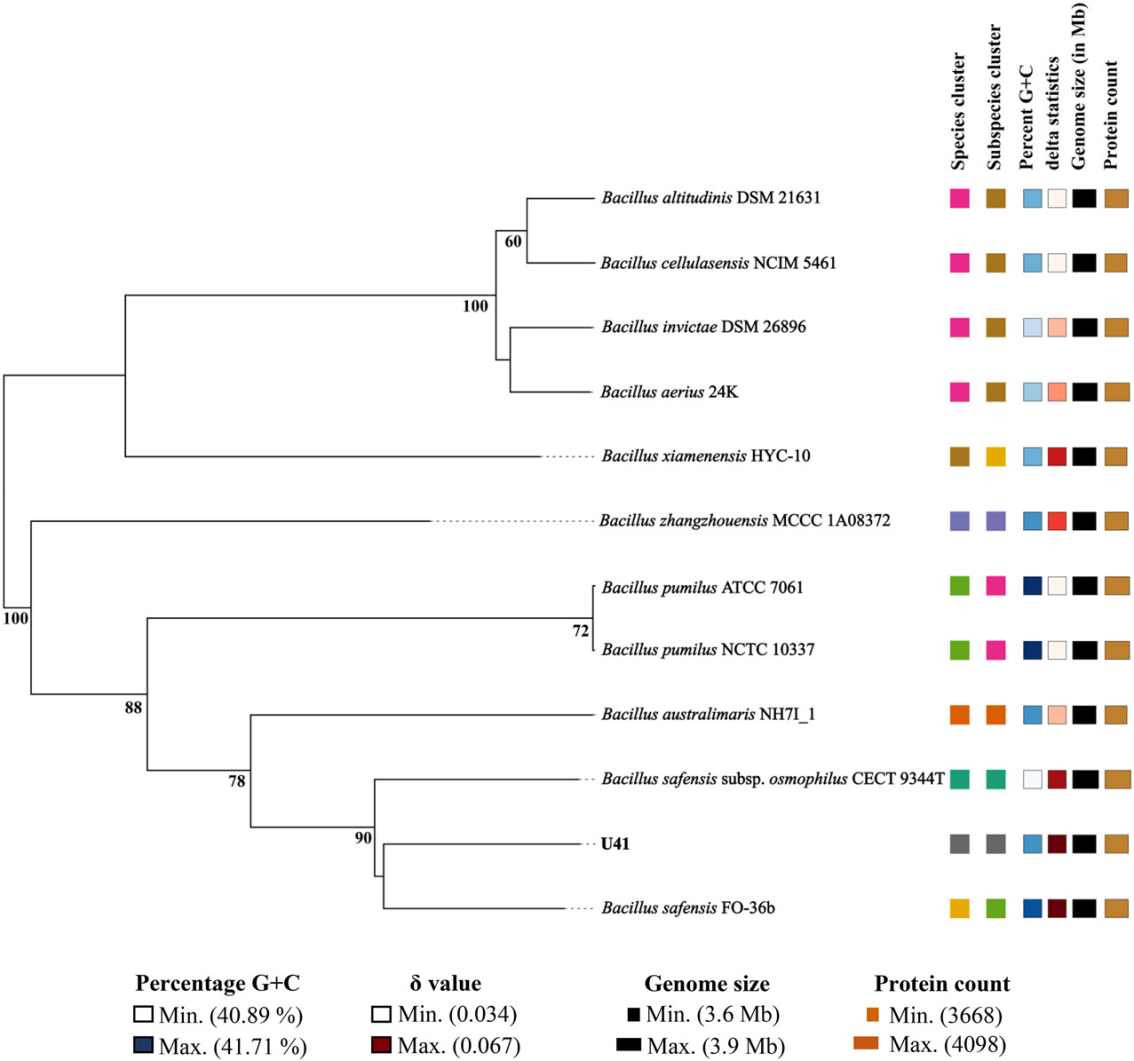
TYGS tree designing based on whole genome sequence inferred with FastME 2.1.6.1 from GBDP distances calculated from genome sequences. The branch lengths are scaled in terms of GBDP distance formula d5. The numbers above branches are GBDP pseudo-bootstrap support values > 60% from 100 replications, with an average branch support of 78.3%. The tree was rooted at the midpoint.

Strain U41 shared more than 97 % similarity of the 16S rRNA gene sequence with several members of the family *Bacillaceae*, which includes 15 species of *Bacillus*. (Supplementary Table S1). Among them, *B. safensis* subsp. *safensis* is the closest phylogenetic relative of strain U41 and shares 99.93 % sequence similarity. The maximum-likelihood tree of the 16S rRNA gene sequence of U41, with all its close relatives, clustered it on the same branch with *B. safensis* species (Fig. 2). A genome-based phylogenetic tree shows the higher bootstrap value (90 %) with the nearest relative species of *B. safensis* (Fig. 3). The digital DNA-DNA hybridization (dDDH) values of strain U41 are 90.8 %, with its closest relative, *B. safensis* species.

Furthermore, functional annotation of the genes by the PANNZER webserver revealed that this isolate might harbor multiple genes associated with cellulase-producing enzymes. The isolate U41 contains 28 genes involved in cellulase production (Table 2).

**Table 2:** Genes involved in cellulase production.

Enzyme	Subtype	No. of gene
Cellulase	Endoglucanase	2
	Exoglucanase	1
	Cellulase	4
	beta-glucosidases	18
	Alpha- glucosidases	1
	oligo-1,6-glucosidases	1
	beta-galactosidases	1
Hemicellulase	Xylanase	8
	Arabinose	6
	Mannose	2
Pectinase	Pectinase	2
	Pectinesterase	3
Ligninase	Laccase, Polyphenol oxidase	2

## 4 Experimental Design, Materials and Methods

*Bacillus safensis* strain U41 was isolated from soils in Santiniketan, West Bengal, India (23.67760 N, 87.68530 E). After the standard dilution method, the strain was obtained by culture on Bushnell Haas agar medium supplemented with 1 % (w/v) CMC (carboxymethylcellulose) medium (BH—CMC). The resulting single colony was sub-cultured by repeated streaking onto BH—CMC agar plates followed by incubation for 48 h at 37 °C [5].

### 4.1 DNA extraction from bacterial culture

Genomic DNA extraction was performed using the QIAgen Blood and Tissue kit (QIAgen Inc., Canada) (Qiagen, Germany) per the manufacturer's guidelines. Quality (OD_260/280_ ratio) and concentration of the isolated DNA were measured using a microplate reader (Agilent BioTek Epoch 2, USA).

### 4.2 Whole genome sequencing and assembly

The whole genome of strain U41 was sequenced using Illumina HiSeq X (2 × 150 paired-end) sequencing technology and generated 7352,576 raw reads. First, raw sequences were visualized in the updated version of FastQC, version 0.11.9 [6], followed by trimming and size selection (>200 bp) in Cutadapt v2.9 [7]. After trimming, 7347,811 paired clean reads were assembled with Unicycler v0.4.4, a hybrid bacterial genomes assembly pipeline with the default setting. The assembly pipeline was optimized and involved an error correction of sequenced reads with BayesHammer [8] and assembly with SPAdes, v3.13.0 [9], with a k-mer value of up to 99. Quality and summary statistics of the assembled genome were generated using the CheckM v1.1.6 and QUAST v.5.1.0. The assembled genome was visualized as a circular map using the CGView server (https://www.cgview.ca) [10] (Fig. 1). Annotation and gene prediction from the assembled genome was performed using prokka v. 1.14.6 (rapid prokaryotic genome annotation).

### 4.3 Phylogenetic tree construction

The 16S rRNA gene sequence was extracted from the whole genome and similarity search using the latest EzBioCloud server (www.ezbiocloud.net) with validated type strains [11]. All available almost complete 16S rRNA gene sequences from closely related type taxa were obtained from the NCBI database (www.ncbi.nlm.nih.gov) and aligned using the MUSCLE algorithm in the MEGA X program [12]. Gaps and missing data were fixed with the complete deletion option, and nucleotide substitution was done using the Tamura 3-parameter with the Gamma distribution evolutionarily invariable model (T92+G+I) [13]. The maximum-likelihood trees were reconstructed using bootstrap values based on 1000 replications in Mega version X (Fig. 2). Whole-genome-based taxonomic analysis, namely genome-to-genome distances (GGDs) and digital DNA-DNA hybridization (dDDH) were performed using the Type Strain Genome Server (TYGS) (https://www.tygs.dsmz.De) [14] (Fig. 3). The whole-genome-based phylogenetic tree was constructed using FastME [15] from the genome blast distance phylogeny (GBDP). The trees were rooted at the midpoint [16].

## Limitations

Not applicable.

## Ethics Statement

The authors have read and follow the ethical requirements for publication in Data in Brief and confirming that the current work does not involve human subjects, animal experiments, or any data collected from social media platforms.

## CRediT authorship contribution statement

**Binoy Kumar Show:** Conceptualization, Data curation, Formal analysis, Methodology, Validation, Writing – original draft. **Andrew B. Ross:** Conceptualization, Investigation, Project administration, Supervision, Writing – review & editing. **Raju Biswas:** Data curation, Formal analysis, Resources, Writing – original draft. **Shibani Chaudhury:** Conceptualization, Investigation, Project administration, Supervision, Writing – review & editing. **Srinivasan Balachandran:** Conceptualization, Investigation, Project administration, Supervision, Writing – review & editing.

## Data Availability

Microbe sample from Bacillus sp. U41 (Original data) (NCBI).

## Appendix Supplementary materials

Image, application 1
