# Supplementary material for: Draft genome sequence data on Bacillus safensis U41 isolated from soils of Santiniketan, India
Source: Data Brief. 2024 May 21;54:110547. doi: 10.1016/j.dib.2024.110547 (PMC11179240; doi:10.1016/j.dib.2024.110547)
Supplement: Supplementary file 1 [file mmc1.docx]

**Supplementary materials to**

***Draft genome sequence data on*** ***Bacillus safensis U41 isolated from soils of Santiniketan, India***

***Binoy Kumar Show^1^, Andrew B. Ross^2^, Raju Biswas^3^, Shibani Chaudhury^1^, Srinivasan Balachandran^1^****

^1^ Department of Environmental Studies, Siksha-Bhavana, Visva-Bharati, Santiniketan 731235, West Bengal, India ([binoyshow@gmail.com](mailto:binoyshow@gmail.com), ORCID ID: 0000-0002-6870-7155; [shibani.chaudhury@visva-bharati.ac.in](mailto:shibani.chaudhury@visva-bharati.ac.in), ORCID ID: [0000-0001-7413-](https://orcid.org/0000-0002-2102-8809)6320; [s.balachandran@visva-bharati.ac.in](mailto:s.balachandran@visva-bharati.ac.in), ORCID ID: 0000-0003-4247-408X)

^2^School of Chemical and Process Engineering, University of Leeds, Leeds, LS2 9JT, United Kingdom

([A.B.Ross@leeds.ac.uk](mailto:A.B.Ross@leeds.ac.uk))

^3^ Department of Environmental Studies, Siksha-Bhavana, Visva-Bharati, Santiniketan 731235, West Bengal, India ([rajubiswas.26041995@gmail.com](mailto:rajubiswas.26041995@gmail.com), ORCID ID: 0000-0002-6131-7015)

****Corresponding author:*** [***s.balachandran@visva-bharati.ac.in***](mailto:s.balachandran@visva-bharati.ac.in)*.*

| Table S1. Ezbiocloud based 16S rRNA gene sequence similarity of strain MR1 with available type strain sequences in the database. | | | | | | |
| --- | --- | --- | --- | --- | --- | --- |
| Sl. No. | Taxon name | Strains names | Pairwise Similarity (%) | Mismatch/Total nt | Completeness (%) | Accession |
| 1 | *Bacillus australimaris* | NH7I_1 | 99.93 | 1/1471 | 100 | JX680098 |
| 2 | *Bacillus safensis* subsp. *safensis* | FO-36b | 99.93 | 1/1471 | 100 | ASJD01000027 |
| 3 | *Bacillus safensis* subsp. *osmophilus* | BC09 | 99.93 | 1/1470 | 99.93201903 | KY990920 |
| 4 | *Bacillus pumilus* | ATCC 7061 | 99.86 | 2/1471 | 100 | ABRX01000007 |
| 5 | *Bacillus zhangzhouensis* | DW5-4 | 99.86 | 2/1471 | 100 | JOTP01000061 |
| 6 | LJIY_s | FJAT-21963 | 99.59 | 6/1470 | 100 | LJIY01000004 |
| 7 | *Bacillus altitudinis* | 41KF2b | 99.52 | 7/1471 | 100 | ASJC01000029 |
| 8 | *Bacillus xiamenensis* | HYC-10 | 99.46 | 8/1471 | 100 | AMSH01000114 |
| 9 | MKZN_s | GM3FR | 99.39 | 9/1471 | 100 | MKZN01000032 |
| 10 | *Bacillus atrophaeus* | JCM 9070 | 97.28 | 40/1471 | 100 | AB021181 |
| 11 | *Bacillus siamensis* | KCTC 13613 | 97.08 | 43/1471 | 100 | AJVF01000043 |
| 12 | *Bacillus subtilis* | NCIB 3610 | 97.01 | 44/1471 | 100 | ABQL01000001 |
| 13 | *Bacillus nakamurai* | NRRL B-41091 | 97.01 | 44/1471 | 100 | LSAZ01000028 |
| 14 | *Bacillus velezensis* | CR-502 | 97.00 | 42/1402 | 95.38043478 | AY603658 |
| 15 | *Bacillus tequilensis* | KCTC 13622 | 96.94 | 45/1471 | 100 | AYTO01000043 |
| 16 | *Bacillus halotolerans* | ATCC 25096 | 96.94 | 45/1471 | 100 | LPVF01000003 |
| 17 | *Bacillus stercoris* | JCM 30051 | 96.94 | 45/1471 | 100 | MN536904 |
| 18 | *Bacillus spizizenii* | NRRL B-23049 | 96.94 | 45/1471 | 100 | CP002905 |
| 19 | *Bacillus mojavensis* | RO-H-1 | 96.87 | 46/1471 | 100 | JH600280 |
| 20 | *Bacillus vallismortis* | DV1-F-3 | 96.87 | 46/1471 | 100 | JH600273 |
| 21 | *Bacillus rugosus* | SPB7 | 96.87 | 46/1471 | 100 | JABUXO010000041 |
| 22 | *Bacillus amyloliquefaciens* | DSM 7 | 96.80 | 47/1471 | 100 | FN597644 |
| 23 | *Bacillus cabrialesii* | TE3 | 96.80 | 47/1471 | 100 | MK462260 |
| 24 | *Bacillus inaquosorum* | KCTC 13429 | 96.80 | 47/1471 | 100 | AMXN01000021 |
| 25 | *Bacillus nematocida* | B-16 | 96.73 | 48/1469 | 100 | AY820954 |
| 26 | AUQZ_s | NSP9.1 | 96.53 | 51/1470 | 100 | AUQZ01000032 |
| 27 | *Bacillus paralicheniformis* | KJ-16 | 96.39 | 53/1470 | 100 | KY694465 |
| 28 | *Bacillus swezeyi* | NRRL B-41294 | 96.39 | 53/1470 | 100 | MRBK01000096 |
| 29 | *Metabacillus idriensis* | SMC 4352-2 | 96.35 | 52/1423 | 96.81139756 | AY904033 |
| 30 | *Bacillus glycinifermentans* | GO-13 | 96.33 | 54/1470 | 100 | LECW01000063 |
| 31 | *Bacillus licheniformis* | ATCC 14580 | 96.19 | 56/1470 | 100 | AE017333 |
| 32 | *Bacillus sonorensis* | NBRC 101234 | 96.19 | 56/1470 | 100 | AYTN01000016 |
| 33 | *Bacillus haynesii* | NRRL B-41327 | 96.19 | 56/1470 | 100 | MRBL01000076 |
| 34 | *Ectobacillus aegiceratis* | 165 | 95.75 | 62/1458 | 100 | MZ389094 |
| 35 | *Bacillus salacetis* | SKP7-4 | 95.72 | 61/1426 | 97.01492537 | LC367333 |
| 36 | *Falsibacillus pallidus* | DSM 25281 | 95.65 | 64/1471 | 100 | QQAY01000036 |
| 37 | *Bacillus aerius* | 24K | 95.64 | 64/1468 | 100 | AJ831843 |
| 38 | *Bacillus gobiensis* | FJAT-4402 | 95.51 | 66/1470 | 100 | CP012600 |
| 39 | *Metabacillus indicus* | LMG 22858 | 95.47 | 66/1458 | 100 | JGVU01000003 |
| 40 | *Metabacillus flavus* | KIGAM252 | 95.47 | 66/1458 | 100 | ON394536 |
| 41 | JAQV_s | SJS | 95.40 | 67/1458 | 100 | JAQV02000010 |
| 42 | *Bacillus capparidis* | EGI 6500252 | 95.24 | 70/1470 | 100 | KY003162 |
| 43 | *Sutcliffiella rhizosphaerae* | JJ-125 | 95.23 | 70/1469 | 100 | ON753733 |
| 44 | CP045403_s | THAF10 | 95.23 | 70/1468 | 100 | CP045403 |
| 45 | *Bacillus mediterraneensis* | Marseille-P2366 | 95.23 | 62/1300 | 88.45892736 | LT630029 |
| 46 | *Metabacillus herbersteinensis* | D-1-5a | 95.21 | 70/1460 | 99.4568907 | AJ781029 |
| 47 | ABCF_s | SG-1 | 95.17 | 69/1429 | 97.21845319 | ABCF01000001 |
| 48 | *Rossellomorea marisflavi* | JCM 11544 | 95.17 | 71/1470 | 100 | LGUE01000011 |
| 49 | LDWH_s | LK16 | 95.17 | 71/1470 | 100 | LDWH01000014 |
| 50 | *Bacillus acidicola* | 105-2 | 95.17 | 71/1469 | 100 | AF547209 |
